# Pharmacological targets of SGLT2 inhibition on prostate cancer mediated by circulating metabolites: a drug-target Mendelian randomization study

**DOI:** 10.3389/fphar.2024.1443045

**Published:** 2024-08-06

**Authors:** Yilong Lin, Yue Zhang, Songsong Wang, Lin Cao, Ruidan Zhao, Xilai Ma, Qiaolu Yang, Liyi Zhang, Qingmo Yang

**Affiliations:** ^1^ Department of Breast Surgery, The First Affiliated Hospital of Xiamen University, School of Medicine, Xiamen University, Xiamen, China; ^2^ School of Medicine, Xiamen University, Xiamen, China; ^3^ Department of Hematology, Xiangya Hospital, Xiangya School of Medicine, Central South University, Changsha, China; ^4^ The First Clinical College of Guangzhou Medical University, Guangzhou Medical University, Guangzhou, China; ^5^ The School of Clinical Medicine, Fujian Medical University, Fuzhou, China

**Keywords:** SGLT2 inhibitor, prostate cancer, uridine, Mendelian randomization, PheWAS

## Abstract

**Background:**

The relationship between sodium-glucose cotransporter 2 (SGLT2) inhibitors and prostate cancer is still unknown. Although these inhibitors can influence tumor glycolysis, the underlying mechanism requires further exploration.

**Methods:**

A two-sample two-step MR was used to determine 1) causal effects of SGLT2 inhibition on prostate cancer; 2) causal effects of 1,400 circulating metabolites or metabolite ratios on prostate cancer; and 3) mediation effects of these circulating metabolites. Genetic proxies for SGLT2 inhibition were identified as variants in the SLC5A2 gene and glycated hemoglobin level (HbA1c). Additionally, positive control analysis on type 2 diabetes mellitus (T2DM) was conducted to test the selection of genetic proxies. Phenome Wide Association Study (PheWAS) and MR-PheWAS analysis were used to explore potential treatable diseases and adverse outcomes of SGLT2 inhibitors.

**Results:**

Genetically predicted SGLT2 inhibition (per 1 SD decrement in HbA1c) was associated with reduced risk of T2DM [odds ratio (OR) = 0.66 (95% CI 0.53, 0.82), *P* = 1.57 × 10^−4^]; prostate cancer [0.34 (0.23, 0.49), *P* = 2.21 × 10^−8^] and prostate-specific antigen [0.26 (0.08, 0.81), *P* = 2.07 × 10^−2^]. The effect of SGLT2 inhibition on prostate cancer was mediated by uridine level, with a mediated proportion of 9.34% of the total effect. In MR-PheWAS, 65 traits were found to be associated with SLGT2 inhibitors (*P* < 1.78 × 10^−5^), and among them, 13 were related to diabetes.

**Conclusion:**

Our study suggested that SGLT2 inhibition could lower prostate cancer risk through uridine mediation. More mechanistic and clinical research is necessary to explore how uridine mediates the link between SGLT2 inhibition and prostate cancer.

## Introduction

Sodium-glucose cotransporter 2 (SGLT2) inhibitors are a type of oral antidiabetic medication that directly removes glucose of the systemic circulation (Heerspink et al., 2016; Brown et al., 2021). Extensive clinical trials have offered strong evidence supporting the positive impact of these interventions on reducing glucose levels, as well as enhancing cardiovascular, heart failure, and renal outcomes (Neal et al., 2017; Wiviott et al., 2019; Cannon et al., 2020; Bhatt et al., 2021). Additionally, emerging evidence indicated their potential of reducing the risk of cancers. A meta-analysis revealed that SGLT2 inhibitors provided a protective effect against cancers compared to a placebo group, with a notable effectiveness observed for dapaglifozin and ertuglifozin (Benedetti et al., 2022). SGLT2 inhibitors were considered as potential anticancer drugs that could slow down the tumor growth of carcinoma expressing SGLT2 (Koepsell, 2017). The expression of SGLT2 has been confirmed at the mRNA level and by immunohistochemistry in prostate cancer (Scafoglio et al., 2015). However, the associations between SGLT2 inhibitors and prostate cancer are controversial and the underlying metabolic mechanism still need further exploring.

Uridine, a pyrimidine nucleoside, is characterized by its high abundance and solubility in the bloodstream. The bioavailability of uridine is crucial for the synthesis of RNA and cell metabolism. For RNA synthesis, uridine 5′-triphosphate (UTP) participates in the process, which is produced by adding a third phosphate group to uridine. For cell metabolism, uracil and beta-alanine engage in the tricarboxylic acid (TCA) cycle, which are the products of the catabolism of uridine (Connolly and Duley, 1999). A study employed nutrient-sensitized genome-wide genetic screens and the Profiling Relative Inhibition Simultaneously in Mixtures (PRISM) growth assay on 482 cancer cell lines, to identify cells capable of tolerating complete glucose deprivation and to elucidate the underlying mechanisms (Skinner et al., 2023). The research revealed a significant upsurge in uridine levels at various stages of prostate cancer, particularly in tissue samples. These findings suggested that cancer cells can harness uridine and its metabolic byproducts for glycolysis, thereby bolstering their growth metabolism, and this capability appears to be widespread. It also suggested that uridine may play a role in the pathogenesis of prostate cancer. As a SGLT2 inhibitor, empagliflozin has been reported to have the potential to upregulate uridine, thereby potentially protecting the kidney (Bangarbale et al., 2022). However, the influence of SGLT2 inhibitors on uridine metabolism remains controversial.

Mendelian randomization (MR) is a relatively vigorous and widely used approach, which uses single nucleotide polymorphisms (SNPs) as genetic instruments to examine the causal association. In this study, genetic colocalization was employed to selected MR instrumental variables that were correlated with drug target mRNA expression (Chen J. et al., 2023; Bakker et al., 2023). The method can be applied to investigate the biological mechanisms of how SGLT2 inhibition affects type 2 diabetes mellitus (T2DM) and prostate cancer (Bouras et al., 2022; Lin et al., 2022). In earlier studies on cardiovascular-related diseases, similar methods have been employed to obtain instrumental variables for exposure and use metabolites as mediators (Xu et al., 2022; Li et al., 2023). Although previous studies have examined the casual association between inflammation factors, lipids, amino acid alterations and prostate cancer, these studies were limited in sample size and the range of metabolites investigated. Therefore, more comprehensive research is needed to explore the relationship between the metabolome and prostate cancer.

In order to investigate the causal link between SGLT2 inhibitors and prostate cancer risk via circulating metabolites or metabolite ratios, we conducted a two-sample, two-step MR study to delve into the hypothetical metabolic pathways that might interconnect the pharmacological action of SGLT2 inhibitors with the development of prostate cancer. The finding would provide insights into the uridine metabolism linking the effect of SGLT2 inhibitors with prostate cancer.

## Materials and methods

### Study design

**FIGURE 1:**
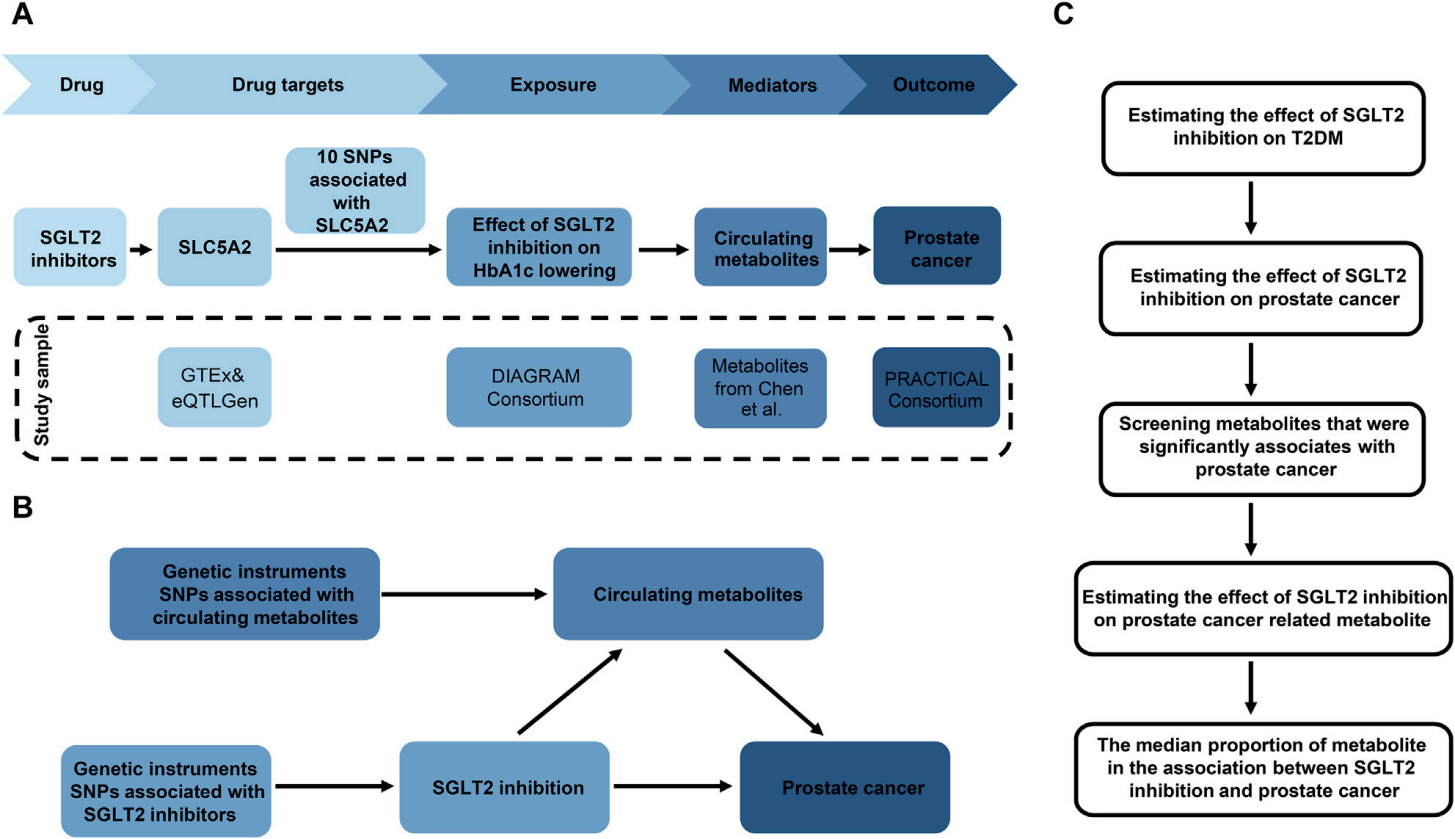
Overview of the study design. **(A)** The flowchart of evaluating the effects of circulating metabolites in mediating the effect of SGLT2 inhibition on prostate cancer. **(B)** The framework of the two-step MR. **(C)** Diagram of the two steps of MR models. HbA1c, glycated hemoglobin; SNP, single nucleotide polymorphism.

The research aims to ascertain if metabolites or metabolite ratios (mediators) play a causal function in facilitating the impact of SGLT2 inhibition on prostate cancer. In this study, we performed a two-sample two-step MR design (Figure 1): Initially, genetic variants representing the effects of SGLT2 inhibition were chosen, followed by the estimation of the causal effects of SGLT2 inhibition on T2DM. Based on that, we further selected prostate cancer as our outcome, and investigating the association between SGLT2 inhibitors and prostate cancer. Then we explored changes in metabolites associated with prostate cancer. In final, we investigated the casual impact of SGLT2 inhibition on prostate cancer related metabolites and calculated mediation effect (Figure 1). The MR analyses fulfilled three core assumptions. First, genetic variants are strongly linked to the outcome. Second, genetic variants are not influenced by other confounding factors. Third, the impact of genetic variants on the outcome is solely driven by exposure. Our study was conducted following the Strengthening the Reporting of Observational Studies in Epidemiology Using Mendelian Randomization (STROBE-MR) guidelines (Skrivankova et al., 2021).

### Data source and genetic instruments for SGLT2 inhibition

The selection of genetic variants serving as proxies for SGLT2 inhibition involved four steps (Figure 1A). 1) Gather GWAS data from the Genotype-Tissue Expression (GTEx) (GTEx Consortium, 2020) and eQTLGen Consortium (Võsa et al., 2021) to identify genetic variants linked to the mRNA expression of SLC5A2 (Supplementary Table S1). 2) Determine the correlation between each SLC5A2 variant and HbA1c levels, which reflect the glucose-lowering impact through SGLT2 inhibition, and identify variants strongly linked to HbA1c (*P* < 1 × 10^−4^). The GWAS data for HbA1c level was obtained from UK Biobank (n = 344,182) (Supplementary Table S1). 3) Investigate if SLC5A2 gene and HbA1c level share the same causal variant through colocalization analysis. Colocalization aims to estimate the posterior probability that SLC5A2 expression and circulating HbA1c levels share the same causal variant in specific region (Zuber et al., 2022). Evidence for colocalization (a posterior probability >70%) was defined as significant (Lin et al., 2023). 4) Conduct a standard clumping process, using the 1,000 Genomes European reference panel to select genetic variants in linkage disequilibrium (LD) with an r^2^ < 0.8 within 1,000 kb. 5) Extract the instrumental variables from the GWAS of HbA1c and obtain the inverse beta values to represent the SGLT2 inhibition.

### Data source and genetic instruments for circulating metabolites

In this study, we systematically obtained GWAS data of 1,091 metabolites and 309 metabolite ratios from 8,299 individuals from the Canadian Longitudinal Study on Aging (CLSA) cohort, and uridine was available, named as Uridine (1) (Supplementary Table S1). The GWAS summary statistics of the 1,400 metabolites or metabolite ratios were publicly available through the GWAS Catalog study (https://www.ebi.ac.uk/gwas/) and the accession numbers for European GWASs: GCST90199621-90201020 (Chen Y. et al., 2023). We also gathered another data through the IEU Open-GWAS Project (ID: ebi-a-GCST90026036) (Panyard et al., 2021) (Uridine2) (Supplementary Table S1). Given the limited number of metabolite-associated SNPs, we select SNPs related to metabolites using a relaxed threshold (*P* < 1 × 10^−5^, r^2^ < 0.1, and a 500 kb distance) at first. To mitigate the effect of weak IV bias and sensitivity analysis, we replicated the MR analysis with a strict threshold. We selected SNPs at conventional GWAS thresholds (*P* < 5 × 10^−8^), and we excluded SNPs in LD, with an R^2^ value greater than or equal to 0.001 and within a 10 mb distance.

### Study outcomes

To ascertain the significance of the selected SGLT2 inhibitor, a positive control analysis was conducted using T2DM. We utilized the latest 2024 meta-analyses data on T2DM, from the DIAbetes Genetics Replication and Meta-analysis (DIAGRAM) Consortium. The data conclude all ancestry and we choose European individuals (36 GWAS, 242,283 T2DM cases and 1,569,734 controls) (Suzuki et al., 2023) (Supplementary Table S1). We obtained publicly available GWAS summary data for prostate cancer from the Prostate Cancer Association Group to Investigate Cancer-Associated Alterations in the Genome (PRACTICAL) Consortium (Schumacher et al., 2018) (79,148 prostate cancer cases and 61,106 disease-free controls, European ancestry) (Supplementary Table S1). Prostate-specific antigen (PSA) data were from INTERVAL study (Sun et al., 2018) (2,994 plasma proteins in 3,301 healthy participants) (Supplementary Table S1).

### Statistical analyses

#### MR estimates of SGLT2 inhibition on T2DM and prostate cancer

Two-sample univariable MR (UVMR) was used to assess the influence of SGLT2 inhibition on T2DM, prostate cancer, and PSA. The MR results demonstrated the impact of SGLT2 inhibition on outcomes. An odds ratio (OR) > 1 indicated an increased risk of outcome, while an OR < 1 signified a reduced risk of outcome occurrence. Mendelian Randomization Pleiotropy RESidual Sum and Outlier (MR-PRESSO) was employed to detect and correct potential horizontal pleiotropy and heterogeneity, with the objective of identifying and address outlier genetic variants (Bowden et al., 2018; Verbanck et al., 2018). Firstly, we employed the inverse variance-weighted (IVW) approach as the main analytical method, as it can provide the most accurate and robust estimates, given the assurance that all genetic variants are valid after screening (Bowden et al., 2016; Bowden et al., 2019). Then we utilized the weighted median and MR-PRESSO method to estimate the MR effects, improving numerical performance and stability.

#### Mediation MR analysis through metabolites or metabolite ratios

Two-step MR was performed to assess how metabolites or metabolite ratios mediated the association between SGLT2 inhibitor use and prostate cancer (Figures 1B, C). We first used UVMR to screen for metabolites significantly correlated with prostate cancer among 1,091 human circulating metabolites and 309 metabolite ratios (β2). Significant MR outputs (0.05/1,400 = 3.57 × 10^−5^) were regarded based on a *P*-value <0.05 Bonferroni-corrected for the number of metabolites and metabolite ratios. Metabolite that remained significant after *P*-value adjustment under both lenient and strict criteria was considered as prostate cancer related metabolite. Then, we utilized UVMR to assess the impact of SGLT2 inhibition on prostate cancer related metabolites (β1). The mediation proportion of significant metabolite in the association between SGLT2 inhibition and prostate cancer was calculated as the product of β1 and β2 divided by the total effect of SGLT2 inhibition on prostate cancer (β3). Then, we further performed multivariable MR (MVMR) to evaluate the effect of uridine on prostate cancer after adjusting for the genetic effect of SGLT2 inhibition (β2′). The mediation proportion was calculated as the product of β1and β2′ divided by β3.

#### Sensitivity analysis

The MR- PRESSO method detects potential outliers and removing these outliers, thus quantifying the presence of pleiotropy during analysis process (Verbanck et al., 2018). The weighted median approach can reliably estimate when over 50% of genetic instruments are valid (Bowden et al., 2016). In MR analysis, we used weighted median and MR-PRESSO approaches to confirm the robustness and reliability of the IVW MR estimates. The effectiveness of the instrument variables was measured using F statistics, with instruments being considered weak if the F statistics were less than 10. The MR-Egger intercept test was used to detect horizontal pleiotropy for MR estimates (Bowden et al., 2015). To assess the heterogeneity between the instruments, Cochrane’s Q test and the global test for MR-PRESSO were computed. The MR analysis and sensitivity analysis were performed utilizing the “TwoSampleMR” and “MRPRESSO” packages in R software (version 4.2.1). Meta-analysis was performed through the Reviewer Manager software (Version 5.3). To account for multiple testing, we considered a two-sided *P*-value that met the Bonferroni corrected *P* threshold as statistically significant. For the impact of SGLT2 inhibition on T2DM and prostate cancer-related outcomes, a *P*-value <0.05 was considered statistically significant.

#### PheWAS analysis

GeneATLAS (http://geneatlas.roslin.ed.ac.uk/) is a comprehensive database utilizing the UK Biobank cohort, encompassing hundreds of traits and millions of variant associations (Canela-Xandri et al., 2018). These associations were computed using 452,264 UK Biobank British white individuals. GeneATLAS includes information on 778 traits (118 quantitative traits and 660 binary traits) and the database allows for querying of GWAS results for 9,113,133 genetic variants and downloading GWAS summary statistics for over 30 million imputed genetic variants, which corresponds to more than 23 billion phenotype-genotype pairs (Canela-Xandri et al., 2018). We searched the GeneATLAS for trait associated with SGLT2 inhibition SNPs. *p* values of traits for each SNP were corrected using the Bonferroni method, setting the threshold for *P* values at 6.43 × 10^−6^ (0.05/7,780), where 7,780 represented the product of the SNP counts (10) and number of traits (778) queried for each SNP. In addition, we performed MR-PheWAS to explore the potential causal relationship between SGLT2 inhibitors and other diseases. The 2,803 outcomes were obtained from FinnGen consortium R5 release data, including 218,792 individuals and 16,962,023 variants (Kurki et al., 2023). The FinnGen project is a large public-private collaboration that combines genome and health data from 500,000 Finnish biobank participants with digital health records from the Finnish National Health Registers (https://www.finngen.fi/en). IVW was used as the main analytical method and we also performed MR analysis by MR-Egger and weighted median method. Significant MR-PheWAS outputs were regarded based on a *p*-value <0.05 Bonferroni-corrected for the number of traits (0.05/2,803 = 1.78 × 10^−5^). For Sensitivity analysis, the MR-Egger intercept test and Cochran’s Q test was employed to identify horizontal pleiotropy and heterogeneity.

## Results

### Causal effects of SGLT2 inhibition on T2DM and prostate cancer

**FIGURE 2:**
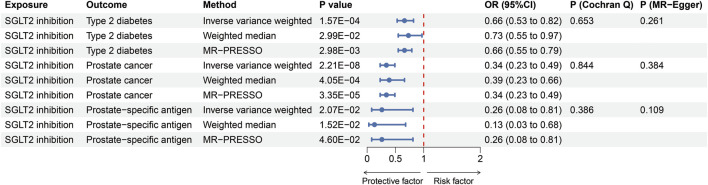
MR estimates of the effect of SGLT2 inhibition on T2DM, prostate cancer and prostate specific antigen. OR, odds ratio; SGLT2, sodium-glucose cotransporter 2.

Altogether, 10 distinct SNPs were chosen as instrumental variables of SGLT2 inhibition. The F-statistics ranged from 15.897 to 97.893, which indicated sufficient instrument strength for univariable analyses (Supplementary Table S2). Through two-sample MR analysis, we observed an association between SGLT2 inhibition and a reduced risk of T2DM [OR = 0.66 (95% CI 0.53, 0.82), *P* = 1.57 × 10^−4^], prostate cancer [0.34 (0.23, 0.49), *P* = 2.21 × 10^−8^] and PSA [0.26 (0.08, 0.81), *P* = 2.07 × 10^−2^], for per 1 SD lowering of HbA1c via SGLT2 inhibition (Figure 2). The weighted median and MR-PRESSO supported these results. No heterogeneity was detected for the impact of SGLT2 inhibition on T2DM, prostate cancer and PSA (*P* = 0.653; *P* = 0.844; *P* = 0.386), and no evidence of horizontal pleiotropy was found (*P* = 0.261; *P* = 0.384; *P* = 0.109) (Figure 2).

### Causal effects of metabolites or metabolite ratios on prostate cancer

**FIGURE 3:**
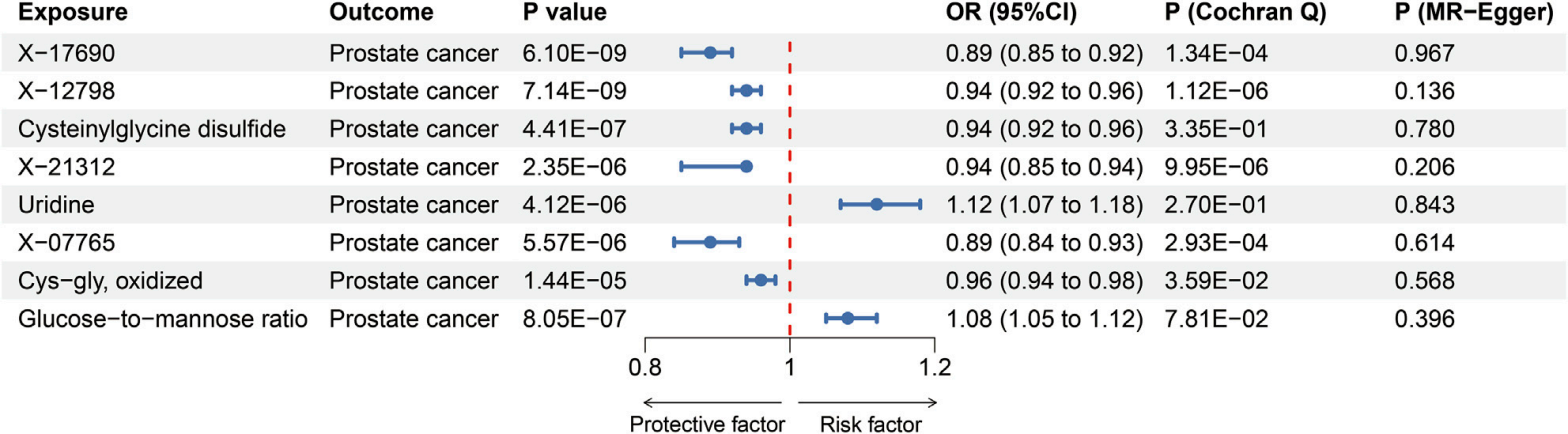
MR estimates of the effects of blood metabolites or metabolite ratios on prostate cancer. OR, odds ratio.

We explored the influences of 1,091 metabolites and 309 metabolite ratios on the risk for prostate cancer under the relaxed threshold (*P* < 1 × 10^−5^) of instrument variables selection, and results demonstrated that total seven metabolites and one metabolite ratios were significantly linked to prostate cancer (Bonferroni-corrected *p*-value threshold = 0.05/1,400 = 3.57 × 10^−5^) (Figure 3). X-17690, X-12798, X-21312, and X-07765 were currently unidentified metabolites. The result revealed that cysteinylglycine disulfide [OR = 0.94 (95% CI 0.92, 0.96), *P* = 4.41 × 10^−7^, Figure 3] and cys-gly, oxidized [0.96 (0.94, 0.98), *P* = 1.44 × 10^−5^, Figure 3] could reduce the risk of prostate cancer. Uridine [1.12 (1.07, 1.18), *P* = 4.12 × 10^−6^, Figure 3] was identified as the only risk metabolite for prostate cancer that remains significant even after adjustment. Then, the strict threshold was used to choose instrument variables. Interestingly, only the uridine levels remained significant after Bonferroni corrected and it was considered prostate cancer related metabolite for further analysis.

### Causal effects of SGLT2 inhibition on prostate cancer related metabolite

**FIGURE 4:**
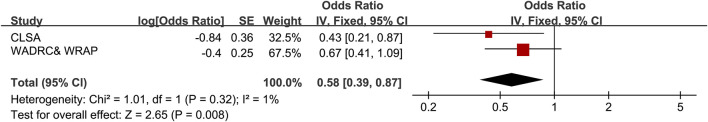
Meta-analysis result of the causal associations between SGLT2 inhibition and uridine levels. CLSA, Canadian Longitudinal Study on Aging; WADRC, Wisconsin Alzheimer’s Disease Research Center; WRAP, Wisconsin Registry for Alzheimer’s Prevention.

The MR results revealed that SGLT2 inhibition decreased the total concentration of uridine (1) [*P* = 0.018, β = −0.84 (95% CI −1.54, −0.14), Table 1]. Cochrane’s Q test implied no evidence of heterogeneity (Q = 7.352, *P* = 0.60, Table 1). To further validate the causal association between SGLT2 inhibition and uridine, we included another GWAS dataset of uridine level and conducted a meta-analysis of the MR results. The meta-analysis indicated that SGLT2 inhibition could decrease uridine levels significantly [OR = 0.58 (95% CI 0.39, 0.87), Figure 4]. Based on previous findings, we discovered that uridine level mediated the reduction in prostate cancer risk associated with SGLT2 inhibition, with the mediating effect accounting for 9.07% of the total effect. In MVMR analysis, SGLT2 inhibitor and uridine were still associated with prostate cancer [OR_SGLT2 inhibitor_ = 0.43 (95% CI 0.24, 0.77), *P*_SGLT2 inhibitor_ = 0.005, OR_uridine_ = 1.13 (95% CI 1.08, 1.18), *P*_uridine_ < 0.001]. And the uridine level had a mediated proportion of 9.34% of the total effect.

**TABLE 1:** MR estimates of the effect of SGLT2 inhibition on uridine level.

Exposure	Outcome	Method	β (95%CI)	*P*	Q	*P*_h_	Intercept	*P*_Int_
SGLT2 inhibition	Uridine (1)	IVW	−0.84 (−1.54, −0.14)	0.018	7	0.601	−0.042	0.987
		WM	−1.02 (−2.00, −0.03)	0.043				
		MR-PRESSO	−0.84 (−1.48, −0.21)	0.031				
	Uridine (2)	IVW	−0.40 (−0.88, 0.08)	0.104	3	0.929	0.008	0.614
		WM	−0.90 (−2.82, 1.02)	0.159				
		MR-PRESSO	−0.40 (−0.70, −0.10)	0.031				

IVW, inverse-variance weighted; *P*h, the *P*-value of Cochrane’s Q test; *P*Int, the *P*-value of MR-Egger intercept test; SGLT2, sodium-glucose cotransporter 2; WM, weighted median.

### PheWAS analysis

We obtained trait associations with prioritized druggable target genes from GeneATLAS, and then we set the threshold for *P* values at 6.43 × 10^−6^ (0.05/7, 780). The top 15 traits with the highest number of SNPs associated with them were listed in Supplementary Table S3. The traits most closely related to SGLT2 inhibitors included platelet count, volume, red blood cell distribution width, reticulocyte count, and systemic impedance. These traits can also serve as potential therapeutic indications and targets for SGLT2 inhibitors. In MR-PheWAS, 65 traits associated with SLGT2 inhibitor (*P* < 1.78 × 10^−5^, Supplementary Table S4) were identified, with 13 of these traits being linked to diabetes. The use of SGLT2 inhibitors was associated with a reduced risk of benign neoplasms of the meninges, pediculosis, acariasis, scabies, strabismus, and venous thromboembolism. On the other hand, the administration of SGLT2 inhibitors might result in various drug side effects, including endometriosis, psoriasis vulgaris, alcoholic gastritis, and other alcohol-related diseases.

## Discussion

In this study, we focused on evaluating the causal associations between SGLT2 inhibition and two main outcomes: T2DM and prostate cancer. Additionally, we explored the metabolites or metabolite ratios related to prostate cancer risk. Our study revealed that genetic variation in SGLT2 inhibition targets was linked to a reduced risk of prostate cancer [0.34 (0.23, 0.49), *P* = 2.21 × 10^−8^] and PSA [0.26 (0.08, 0.81), *P* = 2.07 × 10^−2^]. The circulating uridine level might mediate 9.07% of the impact of SGLT2 inhibition on prostate cancer.

As a part of another glucose import system for cancer cells, SGLT2 is functionally expressed in several cancer types (Scafoglio et al., 2015). Large random clinical trials and meta-analysis have indicated that the medicine which can silence or inhibit SGLT2, such like SGLT2 inhibitors, has the potential ability to suppress cancer cell growth. Research has demonstrated that SGLT2 inhibitors exhibit anti-proliferative effects on liver cancer, breast cancer (Shiba et al., 2018; Komatsu et al., 2020). The mechanisms underlying the inhibition of SGLT2 in prostate cancer remain controversial. Glucose is an indispensable metabolic required for cancer cell survival and growth. SGLT2 inhibitors can block the uptake of glucose by cancer cells, thus whether their anti-proliferative effects on tumor cells are mediated through the reduction of glucose concentration requires further evidence for support. Besides, emerging evidence indicate that SGLT2 inhibitors effect the progression and metastasis of prostate cancer cells through metabolic dysregulation, especially mitochondria function. AMP-activated kinase (AMPK), a master regulator of metabolism, its activation led to reduced cancer cell proliferation (Penfold et al., 2023). A random clinical trial indicated that, Canagliflozin, a kind of SGLT inhibitors, can leads to the activation of AMPK (Hawley et al., 2016; Villani et al., 2016). Act as an attractive strategy based on targeting mitochondrial metabolism, Canagliflozin has expressed potential anti-cancer activity for prostate cancer (Ali et al., 2023). Further research is needed to determine whether SGLT2 affects mitochondrial metabolism through the activation of the AMPK pathway, and more research to investigate whether SGLT2 has other potential effects on metabolic pathways that contribute to its anti-cancer effects.

Our study confirmed that SGLT2 inhibitors reduced the total content of uridine in the serum. This finding highlights the lack of research on the impact of SGLT2 inhibitors on pyrimidine metabolism, particularly regarding uridine levels. One study found no significant difference in uridine levels before and after treatment with canagliflozin on the metabolome of liver cancer cells (*P* = 0.5648) (Nakano et al., 2020). Other studies have mainly discussed the relationship between SGLT2 inhibitors and pyrimidine metabolism, and conclusion remains controversial. An *in vitro* study analyzed the effects of different SGLT2 inhibitors on pyrimidine metabolism (Zügner et al., 2022). The results suggested that treatment with canagliflozin led to an increase in metabolites in the pyrimidine pathway, while the trend was opposite for dapagliflozin and ertugliflozin. An *in vivo* experiment selected 40 type 2 diabetes patients and measured the plasma and urine metabolites after 8 weeks of treatment with a 25 mg dose of the SGLT2 inhibitor empagliflozin. The study results showed that SGLT2 treatment influences pyrimidine metabolism, but changes in UTP levels were not detected (Lu et al., 2022). Further experimental research is needed to investigate whether SGLT2 affects pyrimidine levels in prostate cancer cells and in the plasma metabolites of patients, particularly the influence of uridine.

Given our research findings that SGLT2 inhibitors could reduce uridine levels, and considering that uridine has a positive association with prostate cancer, we hypothesize that the anti-cancer ability of SGLT2 inhibitors may be mediated through uridine. Previous studies have described the characteristics of plasma metabolomics in prostate cancer patients, suggesting a correlation between uridine and PSA metabolism (Markin et al., 2020). Research at the tissue level has confirmed that uridine levels are elevated in prostate tumor tissues compared to adjacent normal control tissues (Ren et al., 2016). The above studies have supported our hypothesis to some extent.

The investigation of whether SGLT2 is worthwhile as a novel anti-pancreatic cancer drug is a promising area for further research. The methods of metabolomics have become more prominent focus for understanding the mechanisms of anti-pancreatic cancer drugs. Proxalutamide is a novel androgen receptor inhibitor currently used in the castration-resistant prostate cancer patients, and it has entered Phase III clinical trials. Research has been conducted to analyze the metabolomics of prostate cancer cells to elucidate the anti-tumor efficacy of Proxalutamide, and results demonstrated that it significantly decreased the intracellular levels of uridine (Qu et al., 2020). Although the study observed significant differences in other metabolites of prostate cancer cells, such as glutamine (a kind of TCA cycle markers), after treatment with Proxalutamide, the results suggest that one of the potential mechanisms of anti-tumor activity of Proxalutamide is the inhibition of pyrimidine synthesis. The study provided support for the potential key role of uridine in the anti-tumor mechanism of SGLT2 inhibitors in prostate cancer. Our study proposes a novel perspective on the potential anti-prostate cancer mechanism of SGLT2 inhibitors, focus on the uridine, providing a theoretical basis for their potential clinical applications in oncology.

Given the potential of SGLT2 inhibitors in preventing prostate cancer, we should not ignore their side effects. A retrospective cohort study found that SGLT2 inhibitors was associated with an approximately three-fold increased risk of genital infections when compared to the use of Dipeptidyl peptidase-4 inhibitors and Glucagon-like peptide-1 receptor agonists (Dave et al., 2019). In addition, SGLT2 inhibitors may lead to other concerns, including cardiovascular safety, acute renal failure, hypoglycemia, volume depletion, volume depletion, euglycemic ketoacidosis, and bone fractures (Scheen, 2019). In our MR-PheWAS analysis, we found SGLT2 inhibitors may lead to some side effects, which provided cautionary notes for the use of SGLT2 inhibitors in the future.

Although previous studies have investigated the impact of SGLT2 inhibitors on prostate cancer using MR, these studies were incomplete and published as research letters. Interestingly, the two studies reported contradictory results: Lin et al. (2024) study suggested a protective effect of SGLT2 inhibitors on prostate cancer, whereas Han et al. (2024) study indicated an increased risk. The reason for the different results obtained from two studies is that the study by Han et al. did not further analyze the instrumental variables’ beta values by taking its opposite into consideration, or convert the OR into its reciprocal as the final outcome. Therefore, the conclusion drawn in Han et al. (2024) study is actually the association between SGLT2 activator and prostate cancer. In comparison to previous research, we used T2DM as a positive control, demonstrating the effectiveness of the obtained SGLT2 inhibitor instrumental variables. The T2DM and prostate cancer GWAS data were sourced from the largest genetic studies to date. What’s more, our study has the novel findings that SGLT2 was associated with prostate cancer mediated by uridine levels. In our investigation of the relationship between circulating metabolites and prostate cancer, we applied the Bonferroni method to correct *P* values, ensuring statistical robustness and the reliability of our research findings. Based on SGLT2 inhibitor’s instrument variables, we also conducted PheWAS analysis to explore the potential causal relationship between SGLT2 inhibitors and other diseases. What’s more, we employed colocalization analysis, a powerful tool in uncovering the pleiotropic effects of certain loci on multiple traits (Zuber et al., 2022). This approach aids in a better understanding of the genetic architecture underlying complex traits. We have provided evidence for the potential anti-cancer mechanism of SGLT2 inhibitors, indicating that the overall concentration of uridine exerts anti-cancer effects on prostate cancer.

Nevertheless, our study is subject to certain limitations. Firstly, the genetic variations emulating SGLT2 inhibition demonstrate the long-term effects of SGLT2 inhibitors, whereas the SGLT2 inhibitors in prior studies are mainly intended for short-term use (Li et al., 2023). Therefore, whether short-term use of SGLT2 inhibitors actually leads to the same results as lifelong effects needs further exploration in experimental and clinical trials. Although the variety of circulating metabolites data we used is comprehensive, due to the lack of available GWAS of pyrimidines in the cohort, limits our ability to further validate the results of our study (Julkunen et al., 2023). Finally, further research is needed to generalize the results of this study to populations other than individuals of European descent.

## Conclusion

In conclusion, this study suggested the causal associations between genetically predicted SGLT2 inhibition, uridine level and prostate cancer. Our findings provide novel insights into the mechanism by which SGLT2 inhibitors reduce the risk of cancer, particularly prostate cancer, and supports further basic and clinical trials of SGLT2 inhibitors in cancer therapy.

## Data Availability

The GWAS Summary statistics used in this study were publicly accessed from the IEU OpenGWAS project (https://gwas.mrcieu.ac.uk/), the GWAS Catalog (https://www.ebi.ac.uk/gwas/), the GTEx Portal (https://www.gtexportal.org/), the eQTLGen Consortium (https://eqtlgen.org/), DIAGRAM consortium (https://diagram-consortium.org/) and PRACTICAL Consortium (http://practical.icr.ac.uk/blog/). Further inquiries can be directed to the corresponding authors.

## Abbreviations

AMPK, AMP-activated kinase; CLSA, Canadian Longitudinal Study on Aging; DIAGRAM, DIAbetes Genetics Replication and Meta-analysis; eQTLs, expression quantitative trait loci; GTEx, Genotype-Tissue Expression; GWAS, genome-wide association study; INTERVEL, Efficiency and safety of varying the frequency of whole blood donation; LD, linkage disequilibrium; MR, Mendelian randomization; MR-PRESSO, Mendelian Randomization Pleiotropy RESidual Sum and Outlier; MVMR, multivariable MR; PRACTICAL, Prostate Cancer Association Group to Investigate Cancer-Associated Alterations in the Genom; PRISM, Profiling Relative Inhibition Simultaneously in Mixtures; PSA, Prostate-Specific Antigen; RNA, Ribonucleic acid; SGLT2, sodium-glucose cotransporter 2; SNPs, single nucleotide polymorphisms; STROBE-MR, Strengthening the Reporting of Observational Studies in Epidemiology Using Mendelian Randomization; T2DM, type 2 diabetes mellitus; TCA, tricarboxylic acid; UTP, uridine 5′-triphosphate; UVMR, univariable Mendelian randomization.
